# Preparation of Ultrafiltration Membrane by Polyethylene Glycol Non-Covalent Functionalized Multi-Walled Carbon Nanotubes: Application for HA Removal and Fouling Control

**DOI:** 10.3390/membranes11050362

**Published:** 2021-05-17

**Authors:** Yu Wang, Mengchan Dong, Xinya Xiong, Xiaoli Gai, Jia Zeng, Guirong Luan, Yufei Wang, Yaochen Wu, Jin Guo

**Affiliations:** National Engineering Laboratory for Advanced Municipal Wastewater Treatment and Reuse Technology, Beijing University of Technology, Pingleyuan No.100, Beijing 100124, China; wangyu@emails.bjut.edu.cn (Y.W.); dongmc@emails.bjut.edu.cn (M.D.); xiongxy@emails.bjut.edu.cn (X.X.); gaixl@emails.bjut.edu.cn (X.G.); zengjia@emails.bjut.edu.cn (J.Z.); lgr19930315@163.com (G.L.); wangyufei-bjut@emails.bjut.edu.cn (Y.W.); wu_yc@emails.bjut.edu.cn (Y.W.)

**Keywords:** low-pressure membrane, multi-walled carbon nanotubes, non-covalent functionalization, polyethylene glycol, membrane fouling

## Abstract

Polyethylene glycol (PEG) non-covalent-functionalized multi-walled carbon nanotubes (MWCNT) membrane were prepared by vacuum filtration. The dispersion and stability of MWCNT non-covalent functionalized with PEG were all improved. TEM characterization and XPS quantitative analysis proved that the use of PEG to non-covalent functionalize MWCNT was successful. SEM image analysis confirmed that the pore size of PEG–MWCNT membrane was more concentrated and distributed in a narrower range of diameter. Contact angle measurement demonstrated that PEG non-covalent functionalization greatly enhanced the hydrophilicity of MWCNT membranes. The results of pure water flux showed that the PEG–MWCNT membranes could be categorized into low pressure membrane. PEG-MWCNT membrane had a better effect on the removal of humic acid (HA) and a lower TMP growth rate compared with a commercial 0.01-μm PVDF ultrafiltration membrane. During the filtration of bovine serum albumin (BSA), the antifouling ability of PEG-MWCNT membranes were obviously better than the raw MWCNT membranes. The TMP recovery rate of PEG–MWCNT membrane after cross flushing was 79.4%, while that of raw MWCNT–_COOH_ and MWCNT membrane were only 14.9% and 28.3%, respectively. PEG non-covalent functionalization improved the antifouling ability of the raw MWCNT membranes and reduced the irreversible fouling, which effectively prolonged the service life of MWCNT membrane.

## 1. Introduction

Low-pressure membrane (LPM) filtration is a membrane separation technology operating under a low pressure (below 1~2 bar), which usually refers to ultrafiltration and microfiltration technology [1,2]. LPM filtration technology can effectively remove particulate pollutants and pathogens from water. Compared to high pressure membrane filtration technology, the energy consumption of LPM is relatively low. As a result, it has been widely used in water supply treatment and wastewater reuse [2]. The increase of operation cost caused by membrane fouling greatly limits the application of LPM [3]. Organic and biological fouling are two most serious membrane fouling phenomena [4], and the former always aggravates the occurrence of biological fouling. Hence, removing organic pollutants or reducing their adhesion is the key to controlling the fouling of LPM.

Carbon nanotubes (CNTs) have attracted great attention since their discovery by the Japanese scientist Sumio Iijima in 1991 [5] for their good mechanical, electrical, and thermal properties. CNTs have great application in the field of biosensors [6,7], high-strength conductive composites [8,9,10,11], and hydrogen storage plant [12]. Meanwhile, due to its good antibacterial [13,14] and adsorption properties [15,16], CNTs are also widely used in the field of water treatment to develop a new generation of inorganic carbon material membrane (CNTs membrane) with high flux and antifouling properties. Anna et al. [17,18] prepared CNTs membrane by vacuum filtration, which could effectively remove virus and bacterial pathogens from water. Nevertheless, the properties of CNTs membrane are limited by the agglomeration of CNTs in aqueous solution, as the hydrophobic characteristics of CNTs tend to promote the aggregation of CNTs. Functionalization of CNTs can improve the hydrophilicity and dispersibility of CNTs in solvent [19,20]. A number of studies have shown that covalent-functionalized CNTs membranes could effectively improve the mechanical properties, hydrophilicity, and pure water flux compared to unmodified CNT membranes. The covalent-functionalized CNTs membranes can effectively enhance the removal of pollutants from water, alleviate membrane pollution, improve the recoverability of the membranes, and extend the service life of the membranes [16,21,22]. Barrejon et al. [16] prepared HCD–SWCNT by cross-linking single-walled carbon nanotubes (SWCNT) with benzidine in the presence of isoamyl nitrite. The HCD–SWCNT membrane prepared by vacuum filtration had obvious selective adsorption ability and recyclability, and could be used to remove organic pollutants from wastewater and realize water/oil separation. Yang et al. [22] covalently functionalized multi-walled carbon nanotubes (MWCNTs) with carboxylic and hydroxylic functional groups, and their corresponding MWCNTs membrane by vacuum filtration. Their results showed that the introduction of functional groups improved the hydrophilicity of MWCNTs, which improved the removal efficiency of HA (>93%) and the service life of the membrane.

Recently, non-covalent functionalization of CNTs received extensive attention as the covalent functionalization of CNTs destroys the inherent properties of CNTs. Non-covalent functionalization of the CNTs is achieved primarily by intermolecular interactions (e.g., Van der Waals’ forces, π–π, hydrogen bond) or by hydrophobic interactions between dispersants and the CNTs, the dispersants are adsorbed or wounded on the CNTs tube walls for functionalization to improve its hydrophilicity and dispersibility in the solution [23]. The method of non-covalent functionalization of CNTs is simple, which only requires ultrasonic mixing of CNTs and dispersants. Sweetman et al. prepared self-supporting SWCNTs membranes by vacuum filtration using non-covalent functionalized SWCNTs by various macrocyclic ligands (derivatized porphyrin, phthalocyanine, cyclodextrin, and calixarene) [24]. The SWCNTs membranes had good electrical conductivity and hydrophilicity, and its mechanical properties were similar to those of the self-supporting CNTs membranes prepared by Triton X-100. Besides, the pure water flux was ten times that of the self-supporting CNTs membranes prepared by Triton X–100. Rashid et al. [25] used biopolymers (bovine serum albumin, lysozyme, chitosan, gellan gum and DNA) to conduct non-covalent functionalization of MWCNTs, and successfully prepared self–supported–MWCNTs nanofiltration membranes by vacuum filtration. It significantly improved the mechanical properties and hydrophilicity of the self-supporting MWCNTs nanofiltration membranes, and had a good effect on the removal of dissolved trace organic compounds and provided a certain desalination capacity.

Polyethylene glycol (PEG) is a non-ionic hydrophilic polyether which is widely used to modify polymer membrane due to its hydrophilicity. Studies show that PEG-modified polymer membrane can effectively alleviate membrane fouling [26,27,28,29,30]. Watchanida et al. [28] prepared modified ultrafiltration poly (ether imide) (PEI) membrane by attaching poly (ethylene glycol) chains onto its surface, and the membrane showed a large improvement in resistance to protein (BSA) fouling. Chen et al. [29] prepared antifouling ultrafiltration membranes by polyacrylonitrile–block–polyethylene glycol (PAN–b–PEG) copolymers through immersion precipitation phase inversion method, and the PAN–b–PEG membranes exhibited better antifouling ability for BSA than origin PAN membranes. Some studies consider using PEG–functionalized carbon nanotubes for membrane modification [31,32]. Wang et al. [31] prepared PEG–g–MWCNTs/PSf hybrid membranes by loading PEG covalent functionalized MWCNTs on polysulfone (PSf) membranes, and their results showed that the hybrid membrane had better antifouling properties. Bai et al. [32] prepared carboxyl functional MWCNTs (MWCNTs–_COOH_) and PEG covalently functionalized MWCNTs (MWCNTs–PEG), and prepared the MWCNTs–_COOH_ and MWCNTs–PEG ultrafiltration membranes by using a UF cell. This study demonstrated that the MWCNTs–_COOH_ and MWCNT–PEG membranes exhibited excellent antifouling performance compared to virgin membrane for all of the NOM foulants (HA, BSA, and SA). As for the PEG-functionalized CNTs, some investigations proved that PEG could reduce the biological toxicity of CNTs [33,34,35,36,37,38]. Sharmeen et al. [33] used PEG covalently functionalized MWCNTs to prepare polyethylene glycol-functionalized MWCNTs/gelatin–chitosan composites. The results indicated that the thermal, swelling, and drug release properties of the MWCNTs/gelatin–chitosan nanocomposites did not show any biological toxicity, and the thermal, swelling, and drug release properties were effectively improved. Adeli et al. [34] reviewed the use of linear polymers to functionalize the surface of CNTs to prepare anticancer drug delivery systems due to their good aqueous solubility and biocompatibility. The results showed that PEG covalent and non-covalent-functionalized CNTs could effectively reduce biological toxicity of CNTs. Lee et al. [35] prepared PEG covalently functionalized and non-covalently coated SWCNTs (SWCNTs–PEG) and found that SWCNTs-PEG could effectively alleviate plasma protein adsorption, and reduce the toxicity of CNTs to organisms. Du et al. [36] found that significant quantity of serum proteins could be quickly adsorbed by CNTs, the binding of serum protein to CNTs may bring some unfavorable effects to organism. This study found that PEG non-covalent functionalization of CNTs could reduce the adsorption of CNTs to serum protein, thus reducing the toxicity of CNTs to organisms. Above studies indicate that both PEG covalent and non-covalent functionalized CNTs can reduce the binding of CNTs to proteins in organisms, and alleviate the biological toxicity of CNTs to organisms [33,34,35,36,37,38]. Until now, some studies use the PEG covalent functionalized MWNTs for membrane modification, while a few investigations consider using PEG non-covalent functionalized MWNTs to prepare MWNTs membrane and performed systematic evaluation on its application in water treatment.

PEG non-covalent functionalization of MWCNT, as a relatively simple CNT modification method, was used in this paper to prepare MWCNT membrane. To evaluate the effectiveness of PEG non-covalent functionalization, a commercial MWCNT–_COOH_ was also selected as a comparison, which represented the covalent-modified MWCNTs. Both MWCNT and MWCNT–_COOH_ were mixed with PEG by ultrasonic treatment to prepare PEG non-covalent-functionalized MWCNTs/MWCNT–_COOH_. Then, PEG non-covalent-functionalized MWCNT or raw MWCNT suspension was loaded onto a PES substrate membrane by vacuum filtration. The objectives of this paper include: (1) evaluation of the effectiveness of PEG non-covalent functionalized MWCNTs membrane, and selection of the suitable molecular weights as the optimal dispersant for non-covalent functionalization of MWCNT; (2) characterization and comparison of the raw MWCNTs membranes with the PEG–MWCNT membranes, and evaluation of their removal efficiency for humic acid (HA). (3) Evaluation of the antifouling performance of PEG–MWCNT membranes and raw MWCNTs membranes during the processing of bovine serum albumin (BSA).

## 2. Materials and Methods

### 2.1. Materials

Multi-walled carbon nanotubes (MWCNT) and carboxyl functionalized multi-walled carbon nanotubes (MWCNT–_COOH_) with an outer diameter of 30–50 nm and length of <10 μm were purchased from Chengdu Organic Chemicals Co., Ltd., Chinese Academy of Science (COCC), Chengdu, China. The MWCNTs were synthesized using chemical vapor deposition and then further purified as reported by the manufacturer, the purity of the received CNTs was 98%. The 0.45 μm PES substrate membranes were purchased from Tianjin Jinteng Experimental Equipment Co., Ltd., Tianjin, China. As a comparison, the commercial 0.01 μm PVDF membranes were purchased from Beijing Amy Ander Membrane Technology Co., Ltd., Beijing, China., which were cut to a circle with an effective filtration area of 13.4 cm^2^. Polyethylene glycol (Mw = 1000 Da, 3350 Da, 6000 Da, 10,000 Da), bovine serum albumin (BSA), and humic aid (HA) were purchased from Sigma-Aldrich (St. Louis, MO, USA). Ethanol (purity 99.7%) was purchased from Beijing Chemical Plant (Beijin, China). The Milli-Q water purification system (Millipore, Boston, MA, USA) was used to obtain ultrapure water at a resistivity of 18.2 MΩ–cm. The ultrapure water was used to prepare the solutions and to rinse the containers. All chemicals used were of analytical grade.

### 2.2. Non-Covalent Functionalization of MWCNT by PEG

Total of 60 mg of MWCNT or MWCNT-_COOH_ was measured with a digital microbalance and dispersed in 110 mL 0.5 % (*w*/*v*) PEG with MW of 1000 Da, 3350 Da, 6000 Da, 10,000 Da, separately. The dispersion was placed in a ice bath and sonicated in ultrasonic crusher (Scientz–950e, Ningbo Xinzhi Biotechnology Co., Ltd., Ningbo, China) for 20 min. The ultrasonic power was 150 W, and pulse duration was 2 s, and pulse delay was 2 s. For the raw MWCNT or MWCNT-COOH suspension, ethanol (110 mL) was used as the dispersant.

### 2.3. Preparation of PEG Non-Covalent Functionalized MWCNT Membrane

**Figure 1:**
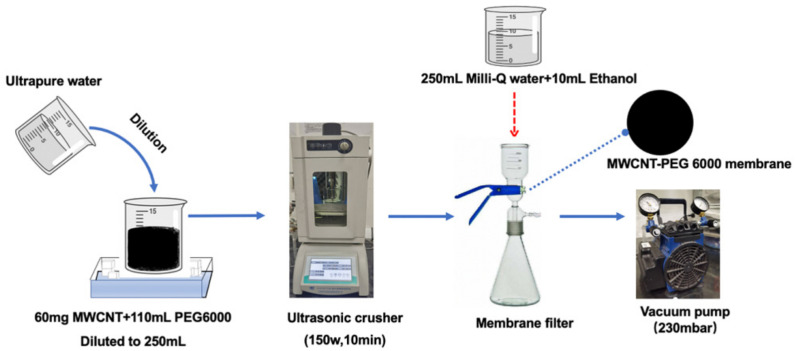
Schematic of preparing PEG non-covalent-functionalized MWCNT membrane.

Before the experiment, the 0.45 μm PES ultrafiltration membranes were soaked in 50% ethanol solution for 2 h to remove the glycerol protective agent from the surface. The membrane was washed multiple times with ultrapure water until the total organic carbon (TOC) of the permeate water approached zero. First, the PEG non-covalent functionalized MWCNT or MWCNT–_COOH_ dispersion prepared in 2.2 was diluted with ultrapure water to 250 mL and the newly obtained dispersion was further sonicated for 10 min in an ice-water batch. Second, 0.45 μm PES membrane was placed on the membrane filter device (Figure 1) and the well-dispersed suspension was filtered through the virgin PES membrane with vacuum of 230 mbar to prepare a MWCNT pre-deposited membrane. Finally, the MWCNT membrane was rinsed with a mixture of 250 mL ultrapure water and 10 mL ethanol solution and then was labeled as PEG–MWCNT/MWCNT–_COOH_ membrane. Raw MWCNT/MWCNT–_COOH_ membrane, which uses ethanol as the dispersant, was obtained with the same operation as above.

### 2.4. Membrane Characterization

Ultraviolet full wavelength spectrum curve of nanotube suspension was measured using a UV–vis–NIR spectrophotometer (U–3900, Hitachi, Tokyo, Japan) within the range of 200–850 nm. Elemental contents of the membranes were analyzed by X-ray photoelectron spectroscopy (XPS, Thermo ESCALAB 250Xi, Boston, MA, USA), including quantitative analysis of C and O elements and the deconvolution of C1 and O1 element peaks. Contact angles of the membranes were measured with a contact angle analyzer (Dataphysics–TP50, Munich, German). At least ten random locations of each membrane were measured to calculate the average contact angle and standard deviation. The surface morphology of raw and PEG-functionalized MWCNT was examined by transmission electron microscopy (TEM, JEM–2100F, Tokyo, Japan). The samples were prepared by dropping the nanotube dispersion onto a carbon-coated copper grid and then dried at room temperature. Scanning electron microscope (SEM) (S–4300, Hitachi, Tokyo, Japan) was used to observe the surface morphology of virgin membranes and MWCNT-modified membranes. The high-power SEM images were obtained at an acceleration voltage of 5 kV. The SEM images were further analyzed by Image J software to obtain apparent pore size distributions of the virgin- and MWCNT-modified membranes. A pressure controlled dead-end filtration unit (Amicon 8050, Millipore, Boston, MA, USA) was used to measure the pure water flux of the membranes, the weighting data of the permeate was automatically recorded by a computer that was connected to an electronic balance.

### 2.5. Permeability of PEG Non-Covalent-Functionalized MWCNT Membrane

In order to accurately evaluate the pure water flux of the virgin membranes and MWCNT-modified membranes, the constant-pressure dead-end filtration system was adopted. By filtering Milli-Q water under pressure of 0.5 bar, 1 bar, 1.5 bar, and 2 bar, respectively, the accumulative permeate volume was automatically weighted and recorded via a data acquisition system. The pure water flux of the virgin membranes and the MWCNT-modified membranes was calculated by linearly fitting the relationship between the permeate flux and transmembrane pressure (TMP).

(1) $$J_{W}=\frac{\Delta m}{\rho \mathrm{AT}\Delta P}$$

where *J_W_* is the permeation flux for pure water (L·m^−2^·h^−1^·bar^−1^); Δm is the weight of ultrapure water collected during a time period (L); ρ is the density of ultrapure water (g/L); A is the effective area of membranes (m^2^); and T is the permeation time interval (h). ΔP is the transmembrane pressure (bar).

### 2.6. Fouling Tests of PEG Non-Covalent-Functionalized MWCNT Membrane

**Figure 2:**
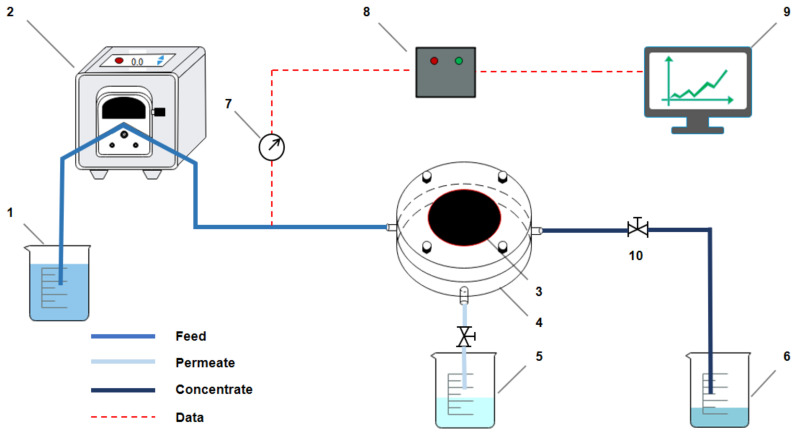
Schematic of the membrane filtration test. 1. Liquid inlet storage tank; 2. peristaltic pump; 3. membrane module; 4. ultrafiltration membrane; 5. liquid outlet storage tank; 6. cross flow liquid storage tank; 7. pressure senor; 8. PLC (programmable logic controller); 9. computer; 10. valve.

Humic acid (HA) and bovine serum protein (BSA) were selected to represent the typical organic pollutants in natural water. A self-made constant-flux dead-end filtration mode with an effective membrane area of 10.17 cm^2^ (Figure 2) was adopted to evaluate the antifouling performance of the PEG non-covalent-functionalized MWCNTs membranes. The membrane filtration system included the membrane filtration unit, an automatic recording and control unit. Prior to the filtration, the membranes were filtered with ultrapure water though the peristaltic pump at a constant flux of 75 L/(m^2^·h) for 30 min, and the pressure was recorded as P_0_. During membrane filtration, the inlet water was replaced with HA/BSA solution and was continuously filtered through the peristaltic pump at a constant flux of 75 L/(m^2^·h), the value of pressure sensor was continuously monitored by the computer and recorded as P. The antifouling performance of the membranes was evaluated by the change of P-P_0_.

In the HA filtration experiment, the feed concentration of HA was 5 mg·L^−1^, and the membrane filtration experiment was continuously operated for 11 h. The samples were taken every 20 min and the sampling time was 10 min. The permeate of HA was measured by UV–vis spectrophotometer at the wavelength of 254 nm. The rejection ratio of HA was calculated with Equation (2):

(2) $$R=(1-\frac{C_{p}}{C_{f}})\times 100\%$$

where R is the rejection ratio of HA (%); C_p_ and C_f_ are the concentrations of permeate and feed HA solutions, respectively.

In the BSA filtration experiment, the feed concentration of BSA solution was 200 mg·L^−1^, the filtration device was operated at a constant flux of 75 L/(m^2^·h) for three cycles. In each cycle, after 85 min BSA filtration, the membrane was cross-flushed with ultrapure water for 6 min. The change of P-P_0_ was used to evaluate the antifouling performance of the membranes.

## 3. Results and Discussion

### 3.1. The Dispersion and Stability of PEG Non-Covalent-Functionalized MWCNT

**Figure 3:**
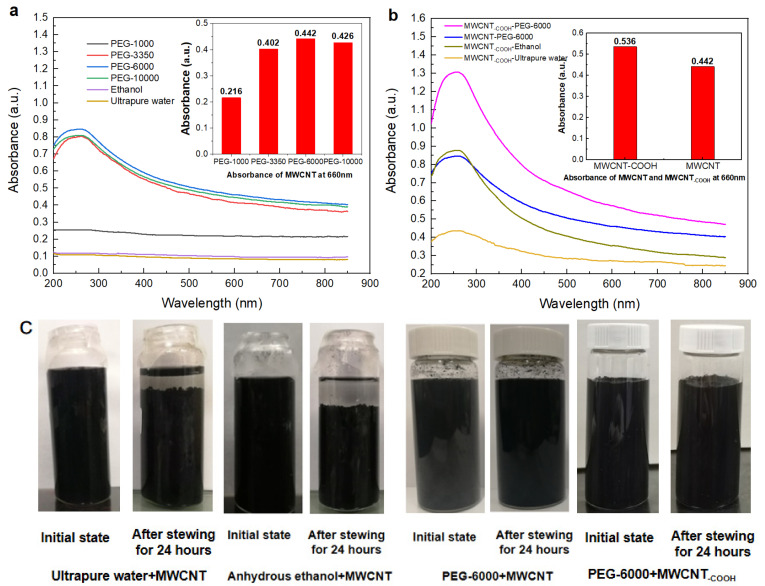
UV–vis–NIR spectrophotometer of PEG non-covalent-functionalized MWCNT dispersions. (**a**) UV–vis–NIR spectrophotometer of non-covalent-functionalized MWCNT dispersion with different chain PEG lengths. (**b**) UV–vis–NIR spectrophotometer of PEG–6000 non-covalent-functionalized MWCNT–_COOH_ dispersion and PEG–6000 non-covalent-functionalized MWCNT dispersion. The illustration shows the absorbance of MWCNT dispersions at 660 nm. (**c**) Stability test of MWCNT dispersions: (left) ultrapure water and anhydrous ethanol as dispersants; (right) PEG–6000 is the dispersant.

The dispersion of the MWCNT non-covalent functionalized with PEG of different molecular weights was measured by UV–vis–NIR spectrophotometer at a wavelength of 660 nm. This wavelength corresponds to an absorption band arising from the van Hove singularities [39]. As shown in Figure 3a, PEG non-covalent functionalization all greatly improved the dispersibility of the MWCNT suspension. The MWCNT solution dispersed by PEG–1000 had the lowest absorbance and the worst dispersibility at 660 nm. In addition, too large molecular weight of PEG would lead to an increase of solution viscosity, which was not conducive to the preparation of membrane. At the same time, the absorbance of PEG–6000 was slightly higher than that of PEG–3350. In conclusion, PEG–6000 was selected to modify MWCNT. As a hydrophilic polymer, PEG could be adsorbed or wound on the walls of MWCNT by blending ultrasound, which improved the hydrophilicity and dispersibility of MWCNT in the solution [40]. As a representative covalent-modified MWCNT, the dispersibility of commercial MWCNT–_COOH_ was compared with PEG–6000 non-covalent-functionalized MWCNT. Figure 3b revealed that the dispersibility of PEG–6000 non-covalent functionalized MWCNT was obviously better than MWCNT–_COOH_ dispersed in ethanol or ultrapure water. The results proved the advantage of the PEG non-covalent functionalized method. We further studied the dispersibility of PEG non-covalent-functionalized MWCNT–_COOH_. As shown in Figure 3b, compared with the PEG–6000 non-covalent-functionalized MWCNT, the UV_660_ absorbance of PEG-6000 non-covalent-functionalized MWCNT–_COOH_ increased by 17.5%, which revealed that the dispersibility of MWCNT–_COOH_ could be further improved by PEG non-covalent functionalization. Besides, as shown in Figure 3c, the dispersions of PEG–6000 non-covalent-functionalized MWCNT and MWCNT-_COOH_ all kept stable in room temperature (22 ℃) for at least 24 h. Compared with the poor stability of MWCNT dispersed in ethanol and ultrapure water, our results proved that PEG–6000 non-covalent functionalization effectively increased both the dispersibility and the stability of MWCNTs. Therefore, PEG with molecular weight of 6000 was selected as the dispersant in the following experiments.

**Figure 4:**
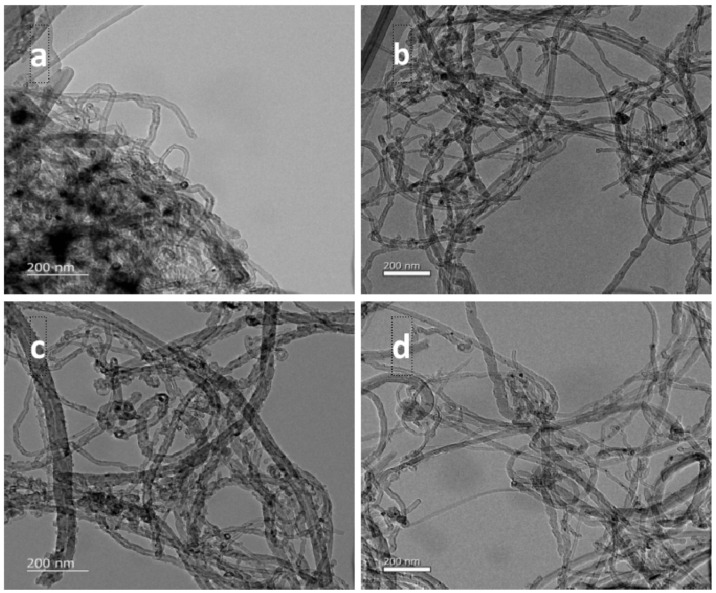
TEM images of PEG non-covalent-functionalized MWCNT. (**a**) raw MWCNT; (**b**) PEG–MWCNT; (**c**) raw MWCNT–_COOH_; (**d**) PEG–MWCNT–_COOH._

The raw MWCNTs/MWCNT–_COOH_ and PEG non-covalent-functionalized MWCNTs/MWCNT–_COOH_ were further observed with TEM. As shown in Figure 4, the surface of raw MWCNT was smooth, while the raw MWCNT–_COOH_ had rough surface which might be related with structure defectiveness. As demonstrated in Figure 4a, raw MWCNT were seriously agglomerated. MWCNT was easy to agglomerate due to their micron length and Van der Waals’ force in the interior [41]. The introduction of carboxylic groups to MWCNT increased its dispersion to a certain extent, as shown in Figure 4c. After non-covalent functionalization with PEG, as displayed in Figure 4b,d, the dispersibility of MWCNT and MWCNT–_COOH_ was greatly improved. The MWCNT were well separated from each other and usually open-ended.

### 3.2. Characterization of PEG Non-Covalent-Functionalized MWCNT Membranes

**Figure 5:**
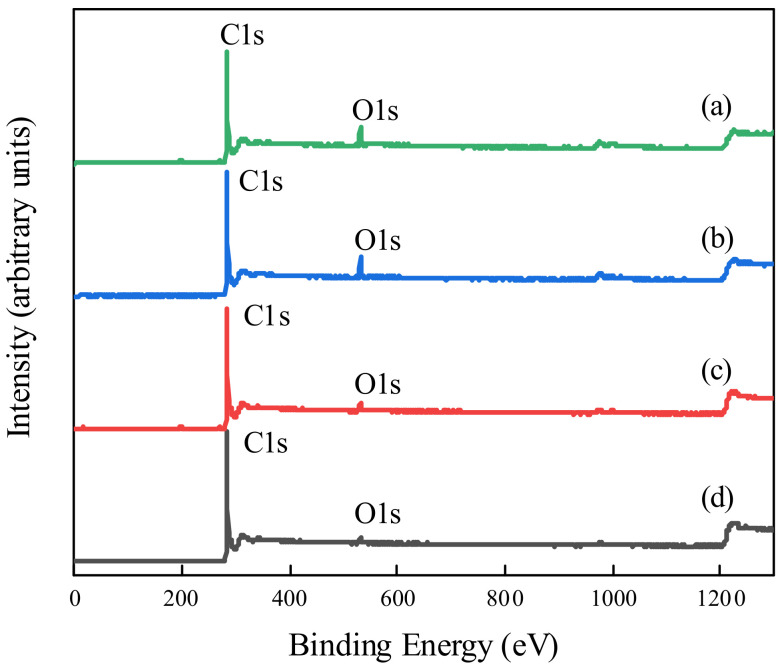
The XPS diagram of PEG non-covalent-functionalized MWCNT membranes: (**a**) PEG–MWCNT–_COOH_ membrane; (**b**) PEG–MWCNT membrane; (**c**) raw MWCNT–_COOH_ membrane; (**d**) raw MWCNT membrane.

**Figure 6:**
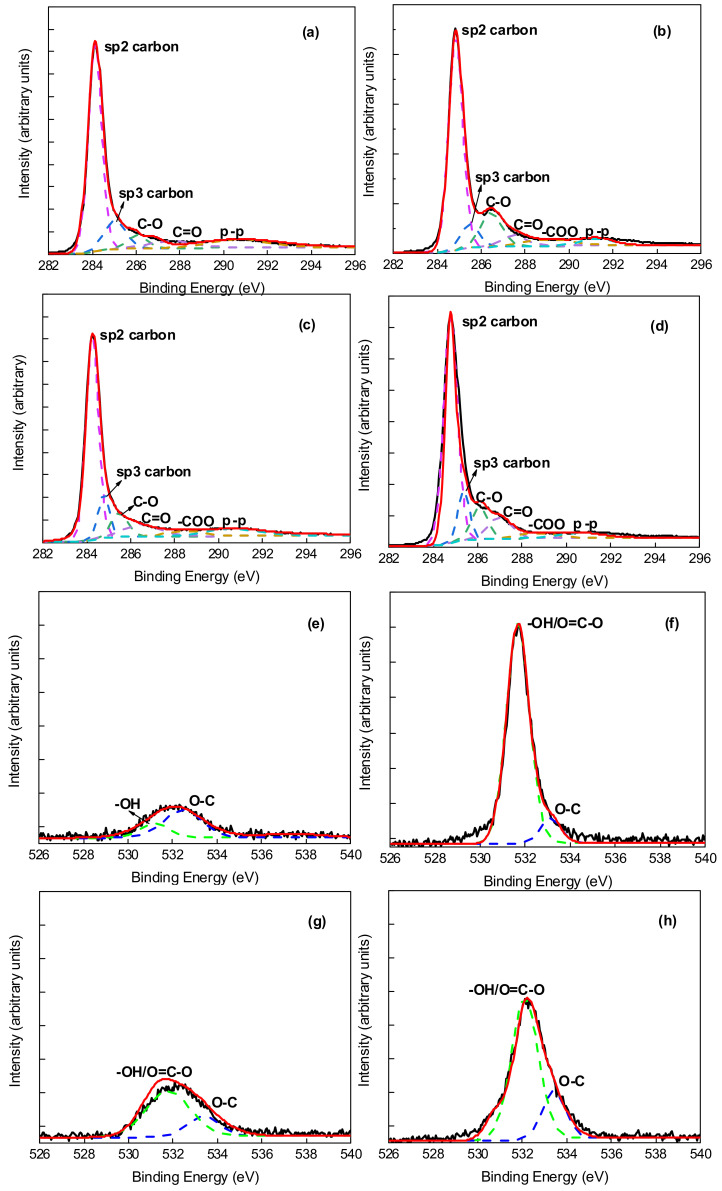
XPS C1s spectrum of (**a**) raw MWCNT membrane; (**b**) PEG–MWCNT membrane; (**c**) raw MWCNT–_COOH_ membrane; (**d**) PEG–MWCNT–_COOH_ membrane; XPS O1s spectrum of (**e**) raw MWCNT membrane; (**f**) PEG–MWCNT membrane; (**g**) raw MWCNT–_COOH_ membrane; (**h**) PEG–MWCNT–_COOH_ membrane.

To characterize and compare the PEG non-covalent-functionalized MWCNT membranes with raw MWCNT membranes, their surface chemical composition and functional groups were analyzed with X-ray photoelectron spectroscopy (XPS). The XPS diagrams of the four membranes are shown in Figure 5. Figure 5a–d display the XPS spectra of PEG–MWCNT–_COOH_ membrane, PEG–MWCNT membrane, raw MWCNT–_COOH_ membrane, and raw MWCNT membrane, respectively. As shown in Figure 5, the elemental survey identified the existence of carbon and oxygen in the four membranes. However, the peak of O1s was significantly higher in the PEG–MWCNT–_COOH_ membrane and PEG–MWCNT membrane than that in the raw MWCNT–_COOH_ membrane and raw MWCNT membrane. The content of C and O elements in the four membranes were also quantitatively analyzed by XPS, as shown in Table 1, the oxygen content of the raw MWCNT membrane and raw MWCNT-_COOH_ membrane were about 2.60% and 3.79%, respectively. After MWCNT and MWCNT-_COOH_ were non-covalent-functionalized with PEG, the oxygen content of the PEG–MWCNT membrane and PEG–MWCNT–_COOH_ membrane was increased to about 5.91% and 7.35%. This result was attributed to the deposition of abundant –OH or O=C–O groups on MWCNT surfaces after PEG non-covalent functionalization. Deconvolution of the XPS C1s (~296–282 eV) and O1s (~540–526 eV) peaks on the four membranes is shown in Figure 6, and the peak assignments were in agreement with the literature [21,42,43]. The C1s was resolved into six peaks and the O1s was resolved into two peaks. For C1s, the peaks at 284.10 eV and 285.00 eV were sp^2^ and sp^3^ hybrid carbon in carbon nanotube, and the peaks at 285.83 eV, 286.98 eV, 288.62 eV, and 290.68 eV were assigned to the C–O, C=O, –COO, and π–π transition, respectively. Combined with the O1s spectrum in Figure 6, which contained –OH or O=C–O at 531.63 eV and O–C at 533.15 eV, the peak intensity of –OH or O=C–O was remarkably increased by the PEG non-covalent functionalization of MWCNT/MWCNT-_COOH_. The XPS data revealed that PEG was successfully introduced into MWCNT/MWCNT-_COOH_-modified membrane by blending with ultrasound. The –OH/O=C–O, O–C contents of the four membranes are also displayed in Table 1.

**Figure 7:**
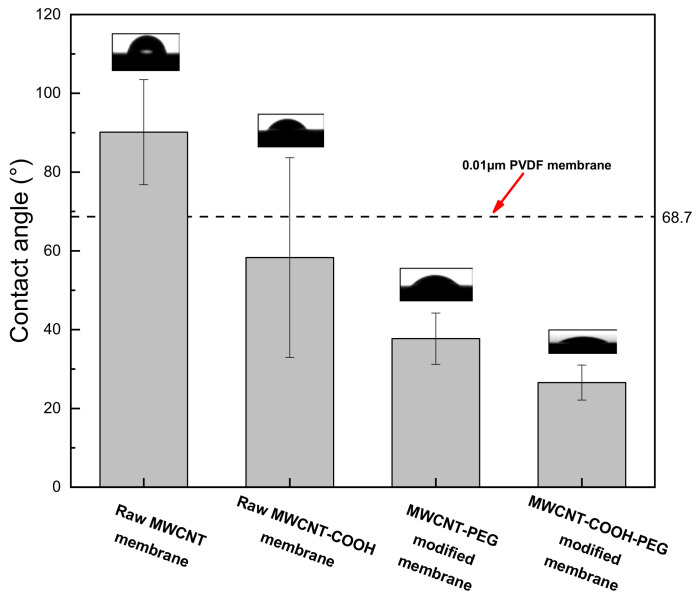
Contact angle of PEG non-covalent-functionalized MWCNT membrane.

Figure 7 demonstrated the contact angle of PEG non-covalent-functionalized MWCNT membrane. As shown, raw MWCNT membrane had higher hydrophobicity with contact angle of 90.1 ± 13.3° compared with the commercial 0.01 μm PVDF membrane (with contact angle of around 68.7°). The contacts angle of raw MWCNT–_COOH_ membrane was 58.3 ± 25.3°, which displayed a relative hydrophilic surface compared with the raw MWCNT membrane. After PEG non-covalent functionalization, the contacts angle of PEG–MWCNT membrane was about 37.7 ± 6.5°, which was much lower than the raw MWCNT membrane, which revealed that PEG non-covalent functionalization had more effectiveness on hydrophilicity increasing compared with the introduction of –COOH onto the surface of MWCNTs. Similarly, the contact angle of PEG–MWCNT–_COOH_ membrane was further decreased to about 26.5 ± 4.4°. Zhang et al. [44] prepared the modified membranes by loading graphene oxide/oxidized carbon nanotubes (GO/OMWCNTs) on the surface of PVDF membranes. The contact angles of the modified membranes decreased due to the incorporation of hydrophilic carbon nanomaterials. Our experiment demonstrated that the hydrophilic PEG could increase the hydrophilicity of the MWCNT/MWCNT–_COOH_ surface after non-covalent functionalization, and decrease the contact angle of the PEG–MWCNT/MWCNT–_COOH_ membranes.

**Figure 8:**
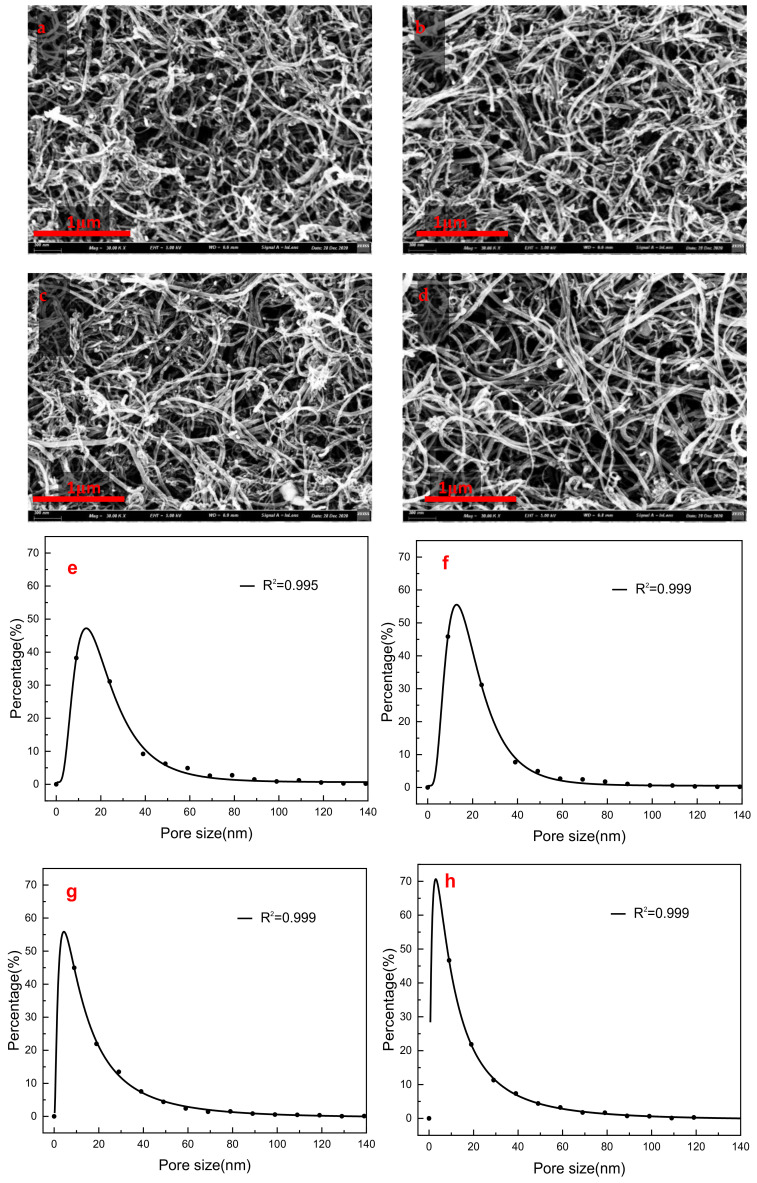
SEM and pore size distribution of PEG non-covalent-functionalized MWCNT membranes. (**a**) and (**b**) SEM images of raw MWCNT/MWCNT-_COOH_ membranes respectively; (**c**) and (**d**) SEM images of MWCNT-PEG and MWCNT–_COOH_-PEG membranes respectively; (**e**) and (**f**) the pore size distribution diagram of raw MWCNT/MWCNT–_COOH_ membranes respectively; (**g**) and (**h**) the pore size distribution of MWCNT–PEG and MWCNT–_COOH_–PEG membranes respectively, the curve in the figure is a fitted lognormal distribution curve.

Figure 8 displays the SEM images and pore size distribution of PEG non-covalent-functionalized MWCNT membranes. It should be noted that the SEM images did not contain any quantitative depth information, so the image analysis results could only be used as the measurement of the apparent pore structure as well as the surface homogeneity of MWCNT membrane. As shown in Figure 8a, raw MWCNTs with ethanol as the dispersant formed a rough and heterogeneous layer on top of the substrate PES membrane, while the surface of PEG–MWCNTs membrane was relatively homogeneous (Figure 8c). Based on quantitative SEM image analysis using ImageJ software, their difference could be further confirmed by the apparent pore size distribution results. As shown in Figure 8e,f, the pore size of raw MWCNT membrane distributed in a wider range compared with that of PEG–MWCNT membrane, which proved that the former had a more heterogeneous structure. As for the membranes fabricated with MWNT–_COOH_ and PEG non-covalent-functionalized MWNT–_COOH_, the latter also presented a more uniform structure, as shown in Figure 8d. Their pore size distribution also confirmed that the pore size of PEG–MWNT–_COOH_ membrane was more concentrated and distributed in a narrower range of diameter (Figure 8h). The apparent average pore size of the four membranes is displayed in Table 2. As shown, there existed a larger standard deviation for the mean pore size, which illustrated that the MWCNT surface had a rather disordered microstructure.

**Figure 9:**
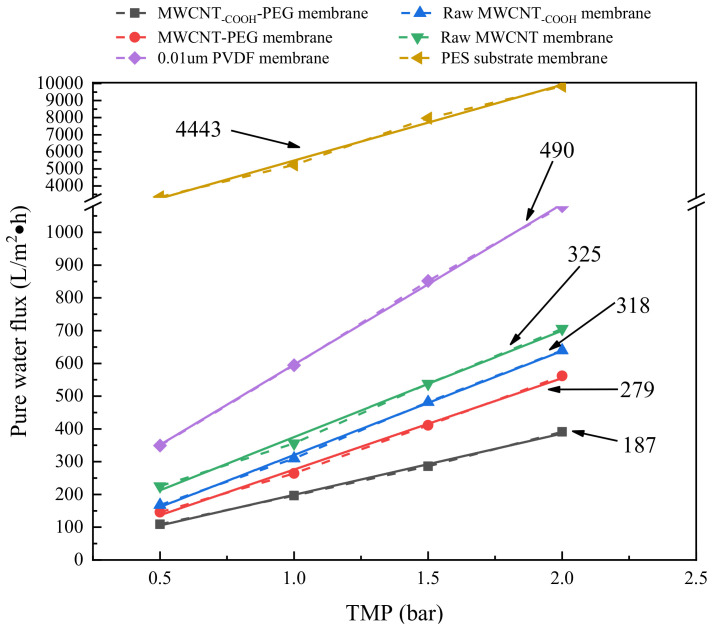
Pure water flux and TMP diagram. In the figure, the dashed line is the connecting line, and the solid line is the linear regression line fitted by experimental data under different membranes. The value beside the line indicates the pure water permeability of the membrane in the unit of L·m·^−2^·h·^−1^·bar^−1^.

The pure water flux of PEG non-covalent-functionalized MWCNT membranes is demonstrated in Figure 9. As shown, the 0.45 μm PES substrate membrane had the highest permeability of 4443 L·h^−1^·m^−2^·bar^−1^. The pure water flux of MWCNT membrane, MWCNT–_COOH_ membrane, PEG–MWCNT membrane, PEG–MWCNT–_COOH_ membrane was 325, 318, 279, and 187 L·h^−1^·m^−2^·bar^−1^, respectively. The commercial ultrafiltration membrane (0.01 μm PVDF membrane) had a pure water flux of about 490 L·h^−1^·m^−2^·bar^−1^. This proved that the four MWCNT-modified membranes could be categorized into low pressure membranes. The transformation of pure water flux proved that the dispersion of MWCNT/MWCNT–_COOH_ was further strengthened after PEG non-covalent functionalization. It helped to form a denser layer with smaller pore size, and caused a relatively lower permeability.

### 3.3. HA Removal by PEG Non-Covalent-Functionalized MWCNT Membranes

**Figure 10:**
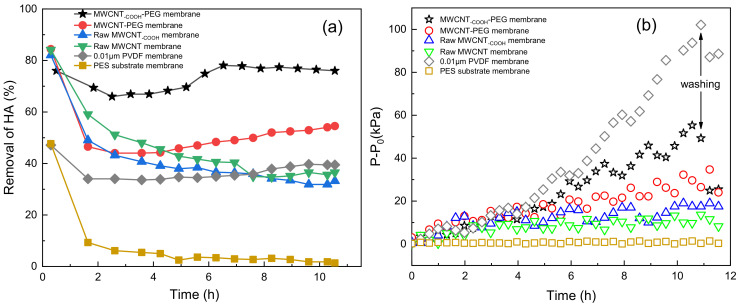
Effects of modified membrane on HA fouling: (**a**) HA removal rate graph; (**b**) P-P_0_ tansformation.

In order to evaluate the effectiveness of PEG non-covalent-functionalized MWCNT membranes on HA removal, a constant flux membrane filtration experiment module was taken to make a systematic investigation. PEG–MWCNT–_COOH_ membrane, PEG–MWCNT membrane, raw MWCNT–_COOH_/MWCNT membranes, and a commercial 0.01 μm PVDF ultrafiltration membrane were compared with each other. Meanwhile, the 0.45 μm PES substrate membrane was also tested for HA removal. During the experiment, the transformation of HA concentration during membrane filtration process was monitored, and the removal rate of HA could be calculated according to Equation (2). The experimental results are demonstrated in Figure 10. Figure 10a,b shows the HA removal rate and the relative TMP (P−P_0_) in the experimental process, respectively.

As displayed in Figure 10a, the HA removal rate of PEG–MWCNT–_COOH_ membrane and PEG–MWCNT membrane was higher than the raw MWCNT–_COOH_/MWCNT membranes. Among the modified membranes, PEG–MWCNT-_COOH_ membrane had the best removal effect on HA, followed by the PEG–MWCNT membrane. With the extension of filtration time, an obvious growing removal trend for HA by the PEG–MWCNT membrane could be observed. The removal rate of HA by the raw MWCNT–_COOH_/MWCNT membranes was as high as 80 % at the beginning, while it rapidly decreased and finally stabilized at about 40%. The 0.45-μm PES substrate membrane could reach 47% HA removal at the beginning, then quickly decreased and finally stabilized at about 2%. This demonstrated that the effect of PES substrate membrane on HA removal was limited. It is obvious that PEG–MWCNT–_COOH_/MWCNT membrane had higher HA removal rate compared to the commercial 0.01 μm PVDF ultrafiltration membrane (40% removal rate). Besides, during the filtration process of HA, relative TMP (P−P_0_) of the commercial 0.01-μm PVDF ultrafiltration membrane rapidly increased to 100 KPa, as shown in Figure 10b. All the MWCNT-modified membranes had a lower TMP increasing. Although the TMP of PEG–MWCNT–_COOH_ membrane also increased to about 50 kPa, the TMP growth rate of PEG–MWCNT membrane was relatively slow. Relative TMP (P−P_0_) of the raw MWCNT–_COOH_/MWCNT membranes also stabilized at a lower level. After cross–flow flushing, the recoverability of PEG–MWCNT–_COOH_/MWCNT membranes were also better than the commercial 0.01 μm PVDF membrane. The influence of PES substrate membrane on the relative TMP (P−P_0_) changes of the four MWCNT membranes could be neglected, as relative TMP (P−P_0_) of 0.45-μm PES substrate membrane stabilized at about 0.5 KPa.

Comparing Figure 10a with Figure 10b, the removal rate of HA by the raw MWCNT–_COOH_/MWCNT membranes gradually decreased and stabilized to about 40%. Their relative TMP (P−P_0_) slowly increased at the beginning and then tend to be stable. As reported, MWCNT has strong adsorption capacity, it has very high removal effect on HA [45,46]. Sabrine et al. [39] prepared porous carbon graphite/multi-walled carbon nanotube composite materials by growing multi-walled carbon nanotubes in situ on porous carbon materials, which effectively improved the adsorption capacity of HA. Yang et al. [21] prepared MWCNT films using functionalized MWCNT for HA removal. The removal rate of HA by MWCNT films could reach 93% by adsorption. We speculate that the adsorption of raw MWCNT–_COOH_/MWCNT membranes plays a major role in HA removal, and HA removal rate could be decreased with the adsorption saturation of raw MWCNT–_COOH_/MWCNT. PEG non-covalent functionalization decreased the active adsorption position on PEG–MWCNT–_COOH_/MWCNT membranes, so the removal rate of HA rapidly reached the lowest point. Nevertheless, the interception effect of PEG-modified membranes further increased the removal of HA. First, compared with the raw MWCNT–_COOH_/MWCNT membranes, the surface of PEG–MWCNT–_COOH_/MWCNT membranes were denser, as shown in the SEM image (Figure 8). Second, the hydrophilicity of PEG–MWCNT–_COOH_/MWCNT membranes enhanced the interaction between the membrane and HA molecules. Moreover, as the surface of PEG–MWCNT–_COOH_/MWCNT membranes was smoother, HA prefers to be removed by the cross-flow flushing rather than adhering on to the membrane surface. Compared with commercial 0.01-µm PVDF membrane, the PEG non-covalent-functionalized MWCNT membrane had better removal efficiency of HA. These results proved that PEG non-covalent-functionalized MWCNT membranes could effectively prolong the service life of membrane and increase HA removal in water.

### 3.4. Antifouling Ability of PEG Non-Covalent-Functionalized MWCNT Membranes

**Figure 11:**
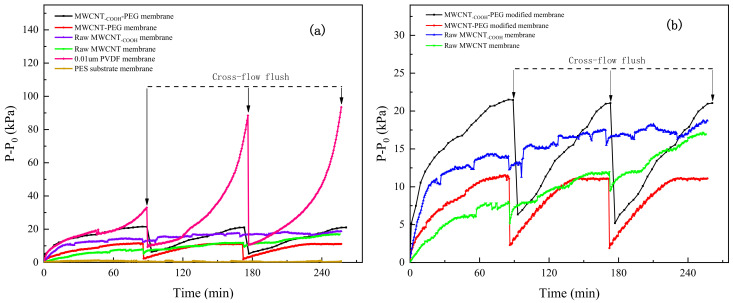
(**a**) TMP growth (P−P_0_) in the constant flux filtration of BSA. (**b**) TMP growth of the four MWCNT membranes in BSA constant flux filtration. The concentration of BSA solution was 200 mg·L^−^^1^ and the flux was stabilized at 75 L·h^−1^·m^−2^. After constant flux filtration of BSA solution for 85 min, cross flow flushing was carried out for 6 min, and each membrane was operated for three cycles.

Figure 11 shows the relative TMP (P−P_0_) change of PEG non-covalent-functionalized MWCNT membranes during the filtration of BSA. The 200 mg·L^−^^1^ BSA solution was filtered at constant flux (75 L·h^−1^·m^−2^) for 85 min, then flushed in cross flow for 6 min, and each membrane was operated for three cycles. As shown in Figure 11a, membrane fouling became severe with the increase of filtration cycles, although membranes could be recovered to some extent through cross flow flush. Relative TMP (P−P_0_) growth of the commercial 0.01-μm PVDF ultrafiltration membrane was the fastest in each cycle, which indicates that the fouling of 0.01 μm PVDF membrane was severe. Compared with commercial 0.01-μm PVDF ultrafiltration membrane, the four MWCNT-modified membranes all improved the antifouling ability of substrate membrane. Figure 11b displayed the relative TMP (P−P_0_) growth of the four MWCNT membranes in BSA constant flux filtration experiments. After three cycles of filtration, the final P−P_0_ of PEG–MWCNT–_COOH_ membrane, PEG–MWCNT membrane, raw MWCNT–_COOH_ membrane and raw MWCNT membrane was 21 KPa, 11 Kpa, 18 Kpa, and 17 KPa, respectively. As shown in Table 3, the TMP recovery rate of PEG–MWCNT membrane after cross flow was 79.4%, followed by the PEG–MWCNT–_COOH_ membrane, in which TMP recovery rate after cross flow was about 70%. As for the raw MWCNT–_COOH_ and MWCNT membrane, the TMP recovery rate after cross flow flush was only 14.9% and 28.3% respectively. PEG modification improved the antifouling ability of the raw MWCNT/MWCNT–_COOH_ membranes, and at the same time, it reduced the irreversible pollution of the raw MWCNT/MWCNT–_COOH_ membranes. The influence of substrate membrane on TMP changes of the PEG-MWCNT membranes could be ignored, as the TMP growth (P-P_0_) of PES substrate membrane was stable at about 0.5 KPa during filtrating BSA.

Some scholars used PEG to modify the surface of ultrafiltration membranes. Du et al. [36] used PEGs with different chain lengths to non-covalently functionalize SWCNT. As the proteins could be adsorbed on the SWCNT with the hydrophobic interactions between the nanotubes and the hydrophobic domains of the proteins, through the adsorption kinetics curves of proteins, it was found that the increase of PEG chain length from 4000 Da to 6000 Da significantly inhibited the adsorption of protein by SWCNTs. Chinpa et al. [28] attached PEG chains onto the surface of asymmetric ultrafiltration polyethyleneimine (PEI) membranes, which improved the hydrophilicity of PEI membranes. Their results of BSA filtration experiment showed that the antifouling performance of modified PEI membrane is much better than that of unmodified PEI membrane. Bai et al. [32] modified PES ultrafiltration membrane with MWCNT, MWCNT–_COOH_, and MWCNT–PEG respectively, and investigated the antifouling performance of membrane with HA, BSA, and SA as model pollutants. Their results showed that the surface of the modified membrane MWCNT-PEG was smoother and hydrophilic, thus enhanced the antifouling ability of the membrane. Our results in Figure 5, Figure 6, Figure 7 and Figure 8 proved that hydrophilic functional groups could be introduced onto the membranes surface after PEG non-covalent functionalization of MWCNT, and the membrane surface became smooth. These all weakened the hydrophobic interaction between protein and MWCNT.

## 4. Conclusions

The non-covalent functionalization of MWCNT by polyethylene glycol (PEG) was successfully carried out. The effect of PEG on the dispersion of MWCNT suspension was obvious. The following conclusions were drawn:

(1) MWCNT non-covalent functionalized with PEG–6000 had the best dispersion effect and the pore size of PEG-MWNT membrane distributed in a narrower range of diameter, which corresponded to a more concentrated membrane surface. Compared with MWCNT and MWCNT–_COOH_ membrane, the oxygen content of PEG–MWCNT and PEG–MWCNT–_COOH_ membrane was increased, which proved that PEG non-covalent functionalization of MWCNT was successful. PEG non-covalent functionalization greatly enhanced the hydrophilicity of the MWCNT membranes. The results of pure water flux showed that the PEG MWCNT membranes could be categorized into low pressure membranes.
(2) All the MWCNT-modified membranes had lower TMP growth rates compared with the commercial 0.01 μm PVDF ultrafiltration membrane during the HA filtration. The PEG–MWCNT–_COOH_ membrane had the best effectiveness on HA removal, while the PEG–MWCNT membrane had the best recoverability. According to the transformation of HA removal rate and TMP, we speculated that the adsorption of raw MWCNT–_COOH_/MWCNT membranes plays a major role in HA removal. Although PEG non-covalent functionalization occupied the adsorption site of MWCNT, the removal of HA would further rely on the interception effect of the PEG–MWCNT membranes. PEG non-covalent functionalization effectively prolonged the service life of PEG–MWCNT membrane.
(3) Compared with the commercial 0.01-μm PVDF ultrafiltration membrane, the antifouling ability of the four MWCNT-modified membranes were improved during the filtration of BSA. The TMP recovery rate of PEG–MWCNT membrane after cross flushing was 79.4%, followed by the PEG–MWCNT-_COOH_ membrane with a TMP recovery rate of 70%. The TMP recovery rates of raw MWCNT–_COOH_ and MWCNT membrane were only 14.9% and 28.3%, respectively. PEG non-covalent functionalization improved the antifouling ability of the raw MWCNT/MWCNT–_COOH_ membranes, and reduced the irreversible fouling of raw MWCNT/ MWCNT–_COOH_ membranes.

## Figures and Tables

**Table 1:** Quantitative analysis of C and O elements by XPS.

Membrane Type	PEG–MWCNT–_COOH_ Membrane	PEG–MWCNT Membrane	Raw MWCNT–_COOH_ Membrane	Raw MWCNT Membrane
C content (%)	92.65	94.09	96.21	97.40
O content (%)	7.35	5.91	3.79	2.60
–OH/O–C=O content (%)	2.47	2.25	0.96	0.32
O–C content (%)	0.41	0.13	0.32	0.42

**Table 2:** Apparent mean pore diameter of PEG non-covalent-functionalized MWCNT membranes.

Type	Pore Size (nm)		Porosity (%)	
	Mean Size	SD	Ratio	SD
Raw–MWCNT	29.0035	27.0679	18.46	0.0518
Raw–MWCNT_–COOH_	23.0691	24.3588	17.44	0.0017
PEG–MWCNT	24.0633	23.9308	19.56	0.0184
PEG–MWCNT_–COOH_	21.9863	20.4326	7.81	0.0910

**Table 3:** The TMP recovery rate after cross flow flush.

Membrane Type	Cycle 1 (%)	Cycle 2 (%)	Average (%)
0.01 µm PVDF membrane	71.9	88.1	80.0
PEG–MWCNT–_COOH_-modified membrane	71.4	76.2	73.8
PEG–MWCNT-modified membrane	79.8	79.1	79.4
Raw MWCNT–_COOH_ membrane	14.7	15.1	14.9
Raw MWCNT membrane	35.6	20.9	28.3

## Data Availability

Not applicable.
